# Measuring therapeutic relationship in the care of patients with haemophilia: A scoping review

**DOI:** 10.1111/hex.12827

**Published:** 2018-08-29

**Authors:** Erin McCabe, Maxi Miciak, Liz Dennett, Patricia Manns, Christine Guptill, Jeremy Hall, Douglas P. Gross

**Affiliations:** ^1^ Faculty of Rehabilitation Medicine University of Alberta Edmonton AB Canada; ^2^ Performance Management and Evaluation Alberta Innovates Edmonton AB Canada; ^3^ John W. Scott Health Sciences Library University of Alberta Edmonton AB Canada; ^4^ Department of Biomedical Engineering University of Alberta Edmonton AB Canada

**Keywords:** blood coagulation disorders, inherited, health‐care surveys, patient outcome assessment, patient participation, patient‐reported outcome measures, professional‐patient relations, validation studies

## Abstract

**Objective:**

We conducted a scoping review of the tools used to measure therapeutic relationship in patients with haemophilia.

**Background:**

Haemophilia is an inherited bleeding disorder caused by a deficiency of a clotting factor in the blood. Therapeutic relationship is foundational to the management of patients with chronic diseases like haemophilia. A reliable and valid measurement tool for assessing therapeutic relationship is needed to evaluate the quality of care received by these patients, and to rigorously study the association between therapeutic relationship and the outcomes of treatment.

**Methods:**

We adopted the Arksey and O'Malley framework for scoping studies. The following electronic databases were searched for studies that measured a construct related to therapeutic relationships in haemophilia care: MEDLINE, EMBASE, CINAHL, PsycINFO and Scopus. We inventoried these studies, identified the measurement tools used, and described each tool by purpose, content, measurement properties and target population. We identified gaps in the current evidence and directions for future research.

**Results:**

There were 253 unique records retrieved in the search, and twenty studies were deemed relevant. Ten measurement tools were identified. None of the tools measured therapeutic relationship as a single entity; however, six tools measured constructs considered part of patient‐provider relationship (eg trust, communication, working alliance). There has been little validation testing of these tools in haemophilia patient populations.

**Conclusions:**

There is a need for a validated tool for measuring therapeutic relationship in the care of patients with haemophilia. This review provides a foundation for future research in this area.

## 1 INTRODUCTION

Haemophilia is an inherited bleeding disorder caused by a deficiency of a clotting factor in the blood. Patients are at a lifelong risk of bleeding into joints and muscles. Recurrent bleeding often results in chronic impairment of musculoskeletal structures and function, leading to pain and disability.^1^ Prevention of this process is a priority in the improvement of health and quality of life of patients with haemophilia. This is accomplished through regular encounters and monitoring by an interdisciplinary haemophilia treatment clinic (HTC), which consists of physicians, nurses, physical therapists and social workers.^2^ Successful management of haemophilia requires that patients actively participate in their care with the HTC. This purposeful partnership of patient and health‐care providers from the HTC is described as the “therapeutic relationship.”

Therapeutic relationship has been consistently associated with treatment outcomes in health research.^3^, ^4^, ^5^, ^6^ Kelley et al^4^ conducted a systematic review and meta‐analysis of randomized controlled trials examining the effect of manipulating patient‐clinician relationships on medical outcomes. A significant effect in favour of the enhanced patient‐provider relationships group was found in the meta‐analysis. The review included studies of populations of patients with complex chronic conditions (eg diabetes, asthma, hypertension, oncology, obesity), which requiring on‐going management similar to haemophilia. In the care of patients with haemophilia, therapeutic relationship is widely acknowledged as a fundamental part of providing care.^7^ It has been suggested that a close partnership between patient and health‐care providers facilitates the dynamic management of haemophilia throughout life through tailored treatment and personalized therapeutic goals.^8^ A number of authors have highlighted the significance of therapeutic relationship in the care of patients with haemophilia. Therapeutic relationship has been the topic of expert narrative reviews exploring the evolution of patient‐provider relationships and patient autonomy,^9^, ^10^ and ideas about therapeutic relationship in contemporary haemophilia care.^11^, ^12^, ^13^ Findings from qualitative studies suggest that patients and health‐care providers consider aspects of patient‐provider relationship to be a key component of haemophilia treatment.^12^, ^14^, ^15^, ^16^, ^17^

At present, there is an emphasis in haemophilia research on understanding the factors that influence patient's degree of adherence to treatment, which is important because patient adherence to treatment is linked with positive outcomes such as reduced pain and improved joint health.^18^, ^19^ Further, preliminary research suggests significant associations between patients' degree of adherence to factor replacement therapy and certain dimensions of therapeutic relationship.^20^ Specifically, patients reporting a higher degree of trust in their haemophilia physician have higher rates of adherence to treatment.^20^ Similarly, a good relationship with a haemophilia health‐care provider has been positively correlated with adherence levels.^21^

As interest in this area of haemophilia research grows, it becomes important to establish a validated and standardized approach to measuring therapeutic relationship. A high‐quality measurement tool will improve the validity of research into the processes and mechanisms by which therapeutic relationships impact outcomes, such as pain, joint health, and quality of life for patients with haemophilia. A standardized approach to measurement will also facilitate comparisons between studies of interventions aimed at improving therapeutic relationship.

Given the importance of a validated tool, and the relevance of studying therapeutic relationship in this population, we conducted a scoping review to provide a comprehensive overview of the research in the area of measurement of therapeutic relationship in the care of patients with haemophilia. Although we focus on research applications of measurement, this review also has implications for evaluating quality of care and assessing the patient's experience of care.

The objectives of our scoping study were to:

1. Locate and inventory the studies that assess therapeutic relationship in haemophilia, and describe the nature and extent of this evidence.
2. Identify the measurement tools that were used, and examine the literature associated with each tool.
3. Summarize the characteristics of the tools that are relevant to researchers when selecting an appropriate measure of therapeutic relationship.
4. Identify knowledge gaps in this area and directions for future research.

## 2 METHODS

### 2.1 Design

We adopted the Arksey and O'Malley^22^ framework for scoping studies. There are five stages in the framework^22^: (a) Identifying the research question; (b) Identifying relevant studies; (c) Selecting studies for analysis; (d) Charting the data; and (e) Collating, summarizing and reporting results. We complemented these stages with the recommendations of Levac et al.^23^ Briefly, Levac et al^23^ emphasize the need for an iterative and team approach to study design, establishing inclusion and exclusion criteria, searching and selecting relevant articles, and identifying key variables for data extraction. We incorporated these recommendations into the methods of this study.

### 2.2 Search strategy

We identified studies that were relevant to our research question through online searches of relevant health databases from their inception to April 2017. These searches were performed with the assistance of an experienced health research librarian at the University of Alberta. The following electronic databases were searched: MEDLINE (Ovid), EMBASE (Ovid), CINAHL (EBSCOhost), PsycINFO (Ovid) and Scopus. Each search strategy was adapted to the various databases as required, and we did not apply any search limits.

There were three concepts in our search strategy: (a) the relationship between a health‐care provider and patient, (b) haemophilia and (c) measurement. For each concept, we included multiple synonyms and key words. Additionally, we searched the reference lists of the articles selected for inclusion and hand‐searched one key clinical journal, *Haemophilia,* from 1998 to April 2017. During this stage, as the researchers became familiar with the literature, the selection criteria were established. An example of the search strategy is included as Appendix 1. The full search strategy is available upon request from the corresponding author.

### 2.3 Study selection

Two members of the research team independently screened the titles and abstracts of the publications retrieved in the database search. Full texts of the potentially relevant articles were acquired and appraised in reference to our study selection criteria. We included peer‐reviewed articles that described the development, testing or use of a measurement tool in a research study to assess or measure therapeutic relationship or related construct, focusing on a population of patients with inherited bleeding disorders and the health‐care providers (from any discipline) involved in their treatment. We included an article if it measured a subcomponent of therapeutic relationship (eg trust, empathy, communication) or a construct that may be considered to contribute to therapeutic relationship (eg patient‐centredness, satisfaction with care, shared decision making). We included self‐report questionnaires (patient or health‐care provider perspective), observer‐rated scales and coding schemes, all modes of administration (eg paper and pencil, computerized or interview). Any discrepancies between reviewers that arose during the review process were resolved through discussion. We used a kappa coefficient to quantify inter‐rater reliability between reviewers.

As the reviewers became familiar with the literature, they noticed that therapeutic relationship was often conflated with other constructs related to clinical encounters and that authors often did not clearly define the construct being assessed. This made it difficult to determine the content of the measurement tools. To address the issue, we added an item content analysis step to our methods (described in the “Data analysis” section below), similar to methods used by Eveleigh et al.^24^ This iterative approach to methods is an advantage of scoping study methodology for an emerging research area like therapeutic relationship, where little is known about the literature prior to starting the study.^23^

A second challenge encountered during study selection related to the definition of “patient satisfaction with care.” This term might refer to patient satisfaction with interpersonal aspects of care, satisfaction with the specific intervention or satisfaction with the outcomes of treatment. We addressed this challenge through discussion within the research team, which resulted in a clearer definition and common understanding to only include studies assessing satisfaction with *interpersonal* aspects of care.

### 2.4 Charting the data

A single reviewer extracted relevant study features, which were determined based on our research question and objectives. We obtained a copy of each measurement tool that was identified.

### 2.5 Data analysis

**Figure 1:**
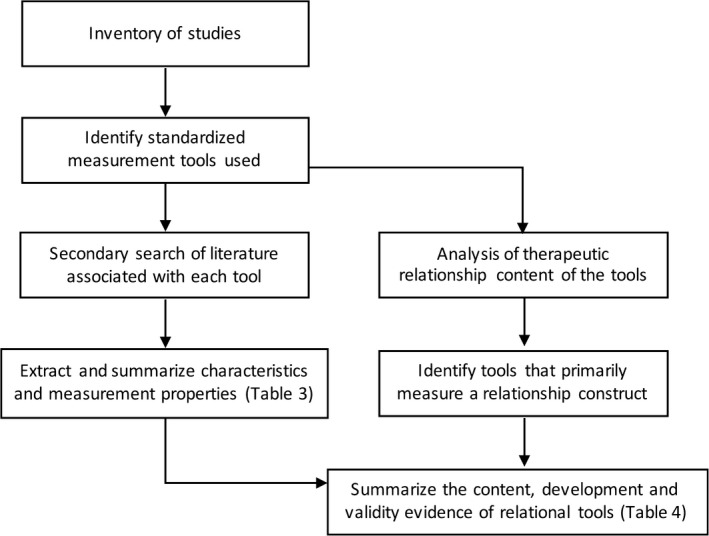
Flow chart of the methods used for data analysis

To describe the nature and extent of the evidence, we calculated descriptive statistics (frequencies and percentages) for the key characteristics of the studies included in the review. Figure 1 shows the flow of the methods of data analysis.

#### 2.5.1 Measurement properties

A useful measurement tool should meet two standards of comprehensiveness.^25^ First, a tool should be accurate and precise through the full range of the variable being measured (eg from poor to strong therapeutic relationships) within the target patient population. It is therefore important to examine the evidence concerning the tool's measurement properties, that is, reliability and validity, in the context of the intended target population.^25^ Second, the content of the tool should adequately represent all the multiple dimensions or components of a health construct.^25^ Therefore, we conducted a second search of the literature to find all published work associated with each measurement tool identified. We searched reference lists, MEDLINE, and the search engine Google, using the name of the tool, any known synonym and abbreviations. We extracted information related to the development and testing of the tool, the measurement properties reported and the theoretical basis of the tools from the articles retrieved in the second search. We examined the extent of the validity evidence for each of the measurement tools identified. We used the COSMIN^26^ (COnsensus‐based Standards for the selection of health status Measurement Instruments) taxonomy and definitions for measurement properties as a guide. We summarized the characteristics of the tools in table form.

#### 2.5.2 Content analysis

The content of the tools was analysed using the framework of therapeutic relationship in physiotherapy developed by Miciak.^27^ Therapeutic relationship has not been conceptualized in the haemophilia literature, and Miciak's framework has qualities that made it appealing for use in this study. The framework was developed using rigorous qualitative methods, it is comprehensive in scope, and it is sufficiently detailed to provide a clear understanding of the fundamental components of the therapeutic relationship.^27^

**Figure 2:**
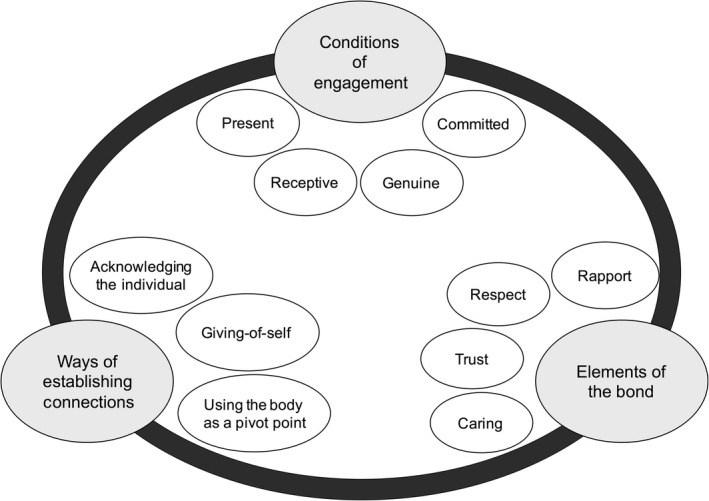
The theoretical framework of therapeutic relationship. There are 3 components in the framework, each with subcomponents which further describe its nature

The three components of therapeutic relationship are as follows: (a) *The conditions of engagement*, (b) *Ways of establishing connections* and (c) *Elements of the bond* (Figure 2).^27^ Further, each component is comprised of subcomponents that describe its nature. *The conditions of engagement* are the attitudes and intentions of the patient and health‐care provider that contribute to “ways of being”—that is, how the patient and health‐care provider “are” together. The *conditions of engagement* are as follows: committed, genuine, receptive and present.^28^ The *ways of establishing connections* describe the actions and behaviours of the health‐care provider and patient within a clinical encounter. *Connections* involve using the body as a pivot point (ie health‐care provider and patient connecting through the patient's body, physiological health condition or physical symptoms), giving‐of‐self and acknowledging the individual (ie validating, individualizing treatment).^29^ The *elements of the bond*—caring, trust, respect and nature of the rapport—describe the emotional or affective resonance between the patient and provider.^27^ Further, Miciak et al identified three themes that should be reflected in the content of a tool intended to measure therapeutic relationship: (a) *Therapeutic relationships are a mutual endeavour*—patients and health‐care providers contribute to the process; (b) *Body is central to the therapeutic relationship*—the patient's experiences with the physiological impact of the health condition (ie body) is the common ground between providers and patients; (c) *Therapeutic relationship is “personal” and “professional”*—positions the therapeutic relationship as part of the health‐care provider's professional responsibilities, while acknowledging the potential for the health‐care provider and patient to have interest and care about the other beyond the clinical reasons for the interaction.^27^

We used the themes and the components in the therapeutic relationship framework to describe the content of the tools and to systematically distinguish the tools that primarily measure a component or subcomponent of therapeutic relationship. We termed these “relational tools,” which we operationally define as a measurement tool that assesses attitudes, intentions, behaviours or feelings between a health‐care provider and a patient. A general patient satisfaction questionnaire is not a “relational tool” as we have defined it. Although it may contain a small proportion of items that address patient‐provider relationship, patient satisfaction questionnaires also typically assess organizational‐ or system‐level health services and processes. We examined the content of a tool using the items as the unit of analysis. We coded each item in reference to the component of therapeutic relationship that it measured (if any). Items that did not fit the therapeutic relationship framework were coded as either “satisfaction with care” or “not interpersonal.” Examples of the item appraisal are included as Appendix 2. For each tool, we calculated the proportion of items in each category (ie relationship, satisfaction or not interpersonal). We distinguished the relational tools based on the proportion of items that measured therapeutic relationship. Finally, we checked whether the tool addressed each of the three themes Miciak identified in therapeutic relationship (personal and professional, body as central and mutuality). Appendix 3 contains the findings of the content analysis. One member of the research team conducted item analysis, and a second member reviewed the results, with any discrepancies resolved through discussion. We summarized the content, function and validity evidence of each relational tool to provide a comprehensive overview of the relational tools used in haemophilia for researchers selecting a measurement tool.

### 2.6 Patient and public involvement and engagement

The aim of patient and public involvement and engagement (PPIE) in this study was to plan, conduct and interpret findings of the research in a manner that was meaningful to patients and their health‐care providers. One patient partner was involved throughout the study as a member of the study team (JH). He is a person with haemophilia and a Master's student at the University of Alberta . He helped design the study, refine the research question and scope, interpret results and critically review written reports. This was accomplished through meetings with the lead researcher, electronic communications and informal conversations at related scientific gatherings. Health‐care providers were also consulted during project planning and after the literature search.

## 3 RESULTS

**Figure 3:**
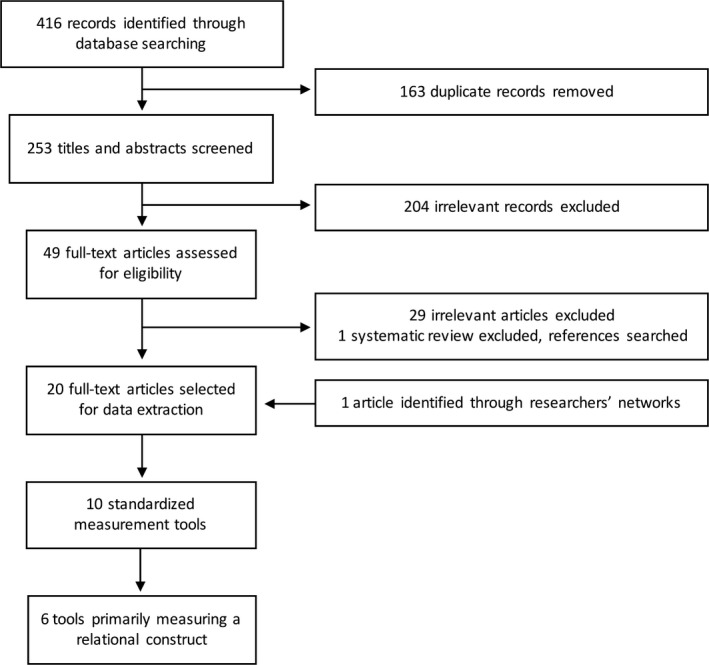
Flow chart of the article search and selection stages

The search and selection process is summarized in Figure 3. The initial search of electronic databases returned 416 records. After 163 duplicates records were removed, two reviewers screened 253 titles and abstracts for potential inclusion. Inter‐rater reliability between reviewers was high in the screening process (Kappa = 0.81). Forty‐nine articles were retrieved for full‐text appraisal. Thirty articles did not fit the criteria for inclusion. One of the articles was a systematic review, which was excluded from further analysis after a search of its reference list for relevant publications. Subsequent to the search, one article was located through the professional networks of the research team. Twenty articles were selected for inclusion, and inter‐rater reliability was good (Kappa = 0.76).

### 3.1 General description of the included studies

The main characteristics of the included articles are summarized in Table 1. The majority of studies (95%) originated in western Europe or North America. A large proportion of the studies (40%) were published in the last 2 years (2016‐2017). The earliest article was published in 1995. A variety of study designs and target populations were used.

**Table 1:** Descriptive characteristics of the studies included in the review

Characteristic	Number of articles	Percentage of studies
Geographic region		
Canada	1	5
United States	5	25
Germany	2	10
Italy	2	10
Spain	4	20
The Netherlands	3	15
Finland	1	5
European (multinational)	1	5
Republic of Georgia	1	5
Date of publication		
2016‐2017	8	40
2011‐2015	3	15
2006‐2010	2	10
2001‐2005	4	20
2000 or before	3	15
Study purpose		
Characterize the haemophilia population	6	30
Evaluate health services	4	20
Evaluative an intervention	4	20
Develop a measurement tool	6	30
Study design		
Cross‐sectional	12	60
Prospective cohort study	2	10
Methodological	6	30
Types of relational constructs assessed		
Working alliance	1	5
Socio‐emotional element	7	35
Communication behaviour	4	20
Satisfaction with health services	8	40
Study population diagnosis		
Haemophilia	15	75
Mixed inherited bleeding disorders	3	15
Mixed haematological conditions	1	5
Haemophilia carriers	1	5
Study population ages		
Adults	5	25
Adult and paediatric patients	8	40
Paediatric patients and parents	3	15
All ages and parents/caregivers	4	20
Disciplines assessed		
Physician	8	40
Nurse	5	25
Physical therapist	3	15
Social worker	3	15
Nonspecific haemophilia health‐care providers	10	50
Other services	2	10

With regard to the relationship construct measured in each study, there were no articles that measured therapeutic relationship as a single entity. One study assessed working alliance—a concept originating in the psychotherapy literature.^30^ Seven studies assessed socio‐emotional elements in therapeutic relationship, for instance, patient trust in the physician, empowerment, collaboration or provider receptiveness. Task‐focused communication—communicative “acts” of the patient or provider—was assessed in four studies. Eight studies evaluated patient satisfaction with health services (n = 8). Six of these articles assessed satisfaction with the services of a HTC, and two assessed satisfaction with other health services (genetic testing, pain therapy). Table 2 contains an inventory of the studies included in this review.

**Table 2:** Inventory of the studies included in the review

Brief citation	Title	Description of study purpose and design	Study population characteristics	Description of relationship construct	Relationship outcome measure(s) used	Measurement properties tested^a^
Ely 1995^42^	The Working Alliance in Pediatric Chronic Disease Management: A pilot study of instrument reliability and feasibility	A pilot study of aiming to test reliability, interpretability and usability of a measure of patient‐provider relationship adapted from the Working Alliance Inventory^60^	Children (7‐14 y old)), adolescents (15‐20 y old), their parents or guardian, physicians and nurse practitioners from a haematology clinic	Quality of the relationship between health‐care providers and their patients	Working Alliance Inventory for Chronic Conditions	Internal consistency, test‐retest reliability, construct validity
Carl 1995^61^	HealthDesk for haemophilia: an interactive computer and communications system for chronic illness self‐management	A pilot study of the implementation of a computer software program for home self‐management. Patient satisfaction with the program, patient‐provider communication and user confidence in self‐management were assessed. Cross‐sectional design	Male patients ages 9‐39 y old with severe haemophilia on home infusion programme (n = 8)	Ease of communication with HTC using the software	Not a standardized measurement tool	None
Jacobson 2016^41^	Telehealth videoconferencing for children with haemophilia and their families: A clinical project	Descriptive study of implementing teleconferencing for evaluating bleeds in children with haemophilia. Data collected regarding bleeds, and user satisfaction. Cross‐sectional design	Male patients (2‐18 y old) with severe haemophilia in New Mexico and Texas, USA (n = 12)	Satisfaction with teleconferencing in terms of communication with haemophilia treatment centre	Not a standardized measurement tool	None
Lock 2016^62^	Optimization of home treatment in haemophilia: effects of transmural support by a haemophilia nurse on adherence and quality of life	A prospective cohort study of the effects of transmural support (home visits) on adherence, quality of life and self‐efficacy for patients with haemophilia	Paediatric patients (mean 9.4 y) with haemophilia A or B, any disease severity, on home infusions (n = 46)	Behaviour of patient in communicating with haemophilia treatment centre	Communication subscale of the Veritas‐PRO	Interpretability
Miesbach 2016^40^	Adherence to prophylactic treatment in patients with haemophilia in Germany	Cross‐sectional survey of German patients to explore associations between adherence to treatment and patient characteristics, such as age, severity of disease, home treatment, pain level, comorbidities, on adherence	Patients with moderate or severe haemophilia A or B, from Germany (n = 397)	Behaviour of patient in communicating with haemophilia treatment centre	Communication subscale of the Veritas‐PRO	None
de Moerloose 2008^21^	A survey of adherence to haemophilia therapy in six European countries: results and recommendations	A descriptive correlational study using structured interviews with patients and health‐care providers. Explored factors that influence adherence, including treatment regimen, environment, patient attitudes, and knowledge of treatment, experiences and preferences	Patients with severe haemophilia A, any age, from six European countries: France, Germany, Italy, Spain, Sweden and the UK (n = 180)	Patient perception of the degree of collaboration between, and relationship with, haemophilia care providers	Not a standardized measurement tool	None
del Río‐Lanza 2016^36^	Information provision and attentive listening as determinants of patient perceptions of shared decision‐making around chronic illnesses	A cross‐sectional survey design, authors used structural equation modelling to describe the pathways of influence of multiple patient‐provider communication variables on patient perceptions of shared decision making	Respondents were adult patients with haemophilia A and B, using regular factor replacement therapy and parents of paediatric patients (n = 181)	Health‐care provider and patient relational communication characteristics, and patient perception of shared decision making	University of Oviedo Survey^b^	Validity, reliability, interpretability
Lamiani 2017^39^	Applying a deliberation model to the analysis of consultations in haemophilia: Implications for doctor‐patient communication	Researchers report on the development of an interaction analysis coding scheme using the Theoretical Model of Deliberation Dialogues. The tool was used to analyse shared decision making during a physician encounter	Patients with haemophilia A over the age of 12, using prophylaxis or on‐demand therapy (n = 30)	Shared decision making dialogue	Theoretical Model of Deliberation Dialogues Coding Scheme	None
Lock 2012^38^	The group medical appointment (GMA) in haemophilia and von Willebrand's disease: A new development in outpatient paediatric care	A prospective cohort study of the implementation of a “group medical appointment” care model. Parents or patients' expectations and experiences of the encounter were collected using a self‐report questionnaire	Families with children (mean age of 8 y), mixed inherited bleeding disorders patients in the Netherlands (n = 53)	Patient expectations and perceptions of health‐care provider communication before and after a health encounter	QUOTE‐Communication Questionnaire	None
Suarez‐Vazquez 2016^37^	Empower Me? Yes, Please, But in My Way: Different Patterns of Experiencing Empowerment in Patients with Chronic Conditions. Health Communication, 0(0), 1‐6	A cross‐sectional survey design, studying the associations between patient and provider communication variables and patients' self‐perception of empowerment were analysed using mixed multiple linear regression modelling	Respondents were adult patients with haemophilia A and B, using regular factor replacement therapy and parents of paediatric patients (n = 181)	Health‐care provider and patient relational communication characteristics and patient empowerment experience	University of Oviedo Survey	Validity, reliability, interpretability
Tran 2016^20^	Physician trust and depression influence adherence to factor replacement: a single‐centre cross‐sectional study	The aim of the study was to explore associations between patient's adherence to factor replacement therapy and demographic, socio‐economic, psychosocial (trust in physician) and health literacy and numeracy variables	Adult patients with moderate or severe haemophilia A or B (n = 91)	Patient's trust in their physician	Wake Forest Trust in Physician Scale	Interpretability
Triemstra 1998^35^	Well‐being of haemophilia patients: A model for direct and indirect effects of medical parameters on the physical and psychosocial functioning	The development and evaluation of a structural equation model for establishing the patient characteristics that impact well‐being in patients with haemophilia	Patients with haemophilia A or B in the Netherlands, aged 15 y or older (n = 980)	Health locus of control, that is, the extent to which individuals perceive health‐care providers to be responsible for their health	Multi‐dimensional health locus of control scale (Dutch adaptation)	Reliability
Arranz 2004^33^	Development of a new disease‐specific quality‐of‐life questionnaire to adults living with haemophilia	Early stages of the development and testing of a haemophilia‐specific quality‐of‐life questionnaire. 75 items were pilot tested, and reduced to 44 items after expert review and pilot testing	Pilot testing of a new questionnaire in adult patients with moderate or severe haemophilia A and B (n = 35)	Patient satisfaction with treatment as a subscale of a disease‐specific health‐related quality of life	Hemofilia‐QoL	Validity, reliability, interpretability
Hacker 2006^34^	A patient satisfaction survey for haemophilia treatment centres	The authors describe the development and testing of a patient‐reported questionnaire to measure satisfaction with the services of their clinic	Paediatric and adult patients with an inherited bleeding disorder from one haemophilia clinic in the United States (n = 271)	Satisfaction with health services including interpersonal skills and quality of care	Mountain States Regional Patient Satisfaction Survey	Validity, reliability, interpretability
Jarvinen 1999^63^	Carrier testing of children for two X‐linked diseases: A retrospective evaluation of experience and satisfaction of subjects and their mothers	The authors used a recall questionnaire to study the experiences of young women who underwent genetic testing as children	Young females from families affected by haemophilia and Duchenne's muscular dystrophy in Finland (n = 23)	Patient perception of their satisfaction and degree of participation in medical decision making	Not a standardized measurement tool	Interpretability
Kalnins 2015^64^	Pain therapy in haemophilia in Germany	A descriptive study based on a questionnaire survey to assess patient's perceptions of pain and pain management	Paediatric and adult patients with haemophilia A or B in Germany (n = 685)	Satisfaction with pain therapy services	Not a standardized measurement tool	None
Kirtava 2005^65^	National haemophilia programme development in the Republic of Georgia	Description of the development of a comprehensive clinic in the Republic of Georgia	Patients with haemophilia A or B from a haemophilia clinic in the Republic of Georgia (n = 104)	Satisfaction with haemophilia treatment centre services	Not a standardized measurement tool	None
Page 2016^66^	Penny wise, pound foolish: an assessment of Canadian haemophilia/inherited bleeding disorder comprehensive care program services and resources	A report on a national survey of Canadian Comprehensive care inherited bleeding disorder programmes and services. Data were collected through interviews with clinicians from haemophilia treatment clinics, and a satisfaction with services survey of Canadian patients	Families and adult patients with any inherited bleeding disorder in Canada (n = 347)	Patient satisfaction with their relationship with their haemophilia treatment centre staff	Not a standardized measurement tool	None
Remor 2005^67^	Psychometric field study of the new haemophilia quality of life questionnaire for adults: The “Hemofilia‐QoL”	A psychometric field study of a newly developed health‐related quality of life questionnaire. The 44‐item draft questionnaire was reduced to 36 items after psychometric evaluation	Patients with haemophilia A and B, mild‐to‐severe disease in Spain (n = 121)	Patient satisfaction with treatment as a subscale of a disease‐specific health‐related quality of life	Hemofilia‐QoL	Validity, reliability, interpretability
von Mackenson 2013^31^	Cross‐cultural adaptation and linguistic validation of age‐group‐specific haemophilia patient‐reported outcome (PRO) instruments for patients and parents	A paper describing the process of translation, and cross‐cultural validation of 3 disease‐specific questionnaires, including one for satisfaction with haemophilia treatment, the Hemo‐SAT	No patients were involved in the language translation study	Satisfaction with haemophilia nurses and specialist services, as a subscale of satisfaction with haemophilia treatment	Hemo‐SAT	Validity, reliability, interpretability

a The measurement properties of the tool which were tested in the study.

b This tool was not named in the studies; therefore, in this paper we have referred to it by the institution at which it was developed.

The aims of the studies were grouped into three categories: (a) seeking to explain interpersonal phenomena in patient care, (b) evaluating an intervention and (c) describing health services. Six studies were assigned to category 1, and these explored the associations between patient and provider characteristics, environmental factors and outcomes of treatment. The four studies in category 2 sought to evaluate an intervention, for example, a new application of a technology or service delivery model. Finally, the four studies in category three aimed to describe health services for patients with haemophilia. The remaining six studies aimed to develop a measurement tool. We identified shared decision making and adherence to treatment as two topics that were frequently studied relative to subcomponents of therapeutic relationship. Five studies were conducted for the purpose of understanding elements of shared decision making. Four studies were aimed at understanding the factors related to adherence to treatment in haemophilia.

### 3.2 Description of measurement tools

Thirteen of the twenty articles described a standardized measurement tool. Ten unique tools were identified: the “Specialist/Nurses” subscale of *Hemo‐SAT,* 31 the “treatment satisfaction” domain of *Hemofilia‐QoL,* 32, 33 *Mountain States Regional Haemophilia and Thrombosis Center Patient Satisfaction Survey,* 34 *Multi‐dimensional Health Locus of Control Scale,* 35 *University of Oviedo Survey* 36, 37 (UOvS), *QUOTE‐Communication Questionnaire* 38 (QUOTE), *Theoretical Model of Deliberation Dialogues* 39 (TMDD), the “Communication” subscale of the *Veritas‐PRO,* 40, 41 the *Wake Forest Trust in Physician Scale* 20 (WFTPS) and the *Working Alliance Inventory for Chronic Conditions* 42 (WAI‐CC).

An additional 27 articles were found that reported on a tool's development or testing. Within the associated literature, we found evidence for all tools regarding content validity and interpretability. Additionally, we found that internal consistency (coefficient alpha) had been reported for all the self‐report questionnaires. The measurement properties of six tools were tested in a haemophilia patient population. Key characteristics of the tools and their associated literature are described in Table 3.

**Table 3:** Description of the measurement tools identified from studies involving patients with haemophilia

Measurement tool	Description	Discipline^a^	Measurement construct^b^	Subscales or domains	Number of items and response scale
Hemofilia‐QoL^32^, ^33^	A disease‐specific quality of life questionnaire, with a “treatment satisfaction” subscale	Haemophilia health‐care providers	2‐item subscale measuring satisfaction with care	Eight domains: physical health, physical role, joint damage, pain, treatment satisfaction, emotional functioning, mental health and social support	2 items, 5‐point Likert scale
Hemo‐SAT specialist/nurses subscale^31^	The Hemo‐SAT was developed to assess patient satisfaction with haemophilia treatment. It contains a subscale, “satisfaction with specialist/nurses”	Physicians and nurses	Satisfaction with care from haemophilia specialists and nurse	Seven domains: ease/convenience, efficacy, burden, side‐effects, specialist/nurse, centre/hospital, general satisfaction	7 items, 5‐point Likert scale
Mountain States Patient Satisfaction Survey^34^	A questionnaire survey designed to measure patient satisfaction with the care provided by a haemophilia treatment centre	Haemophilia health‐care providers	Patient satisfaction services of a haemophilia treatment centre	Four domains: technical competence, interpersonal skills, quality of care and access	37 items, 5‐point Likert scale
Multi‐dimensional health locus of control scale^35^	Measures the degree to which a person perceives others (health‐care professionals) to have control over their health own health	Physician	Patient's health locus of control	Three dimensions: self, others, luck; “Other” measures the extent to which an individual perceives others to be responsible for his or her health	18 items, 6‐point Likert scale
QUOTE‐Communication Questionnaire^38^	A patient‐reported satisfaction questionnaire, based on the theory that patient satisfaction is a function of patient expectations and experiences	Health‐care providers	Patient satisfaction with communication during a health‐care encounter	Two subscales (previsit and postvisit) each with two domains (biomedical and psychosocial)	2 sets of 10 items, 5‐point Likert scale
Theoretical Model of Deliberation Dialogues coding scheme^39^	An observer‐rated coding scheme to analyse shared decision making between patient and physician during a consultation	Physicians	Patient and physician interactions	Codes based on three stages in shared decision making: opening stage (topic or problem introduced), argumentation stage (solutions proposed, arguments for or against a proposal), closing stage (explicit agreement by one or both participants)	A rating scale of Complete or Incomplete; codes describe qualities of an incomplete dialogue
University of Oviedo Survey^36^, ^37^	A patient‐reported questionnaire developed for the purpose of developing statistical models to describe relationships between patient‐provider communication variables	Haemophilia health‐care providers	Patient‐provider communication, patient perceptions of shared decision making and empowerment experiences	Eight subscales: patient participation, patient impact, meaning, health‐care professionals' information provision, emotional support, attentive listening, trust;' patients' collaboration	29 items, 5‐point Likert scale
Veritas‐PRO communication subscale^40^, ^62^	A patient‐reported questionnaire to assess patient adherence to prophylaxis regiment. It includes six subscales, one of which is “communication”	Haemophilia health‐care providers	Communication behaviours of the patient and their haemophilia treatment centre	6 subscales: time, dose, plan, remember, skip, communicate	4 items, 5‐point Likert scale
Wake Forest Trust in Physician Scale^20^	A patient‐reported questionnaire to assess trust in physician	Physicians	Interpersonal Trust	Four domains: fidelity, competence, honesty, global trust	10 items, 5‐point Likert scale
Working Alliance Inventory—Chronic Conditions^42^	A tool to assess patient‐provider relationships in paediatric haematology. 20 forms were designed to assess from the perspective of adolescents, children, their parents, the physician and nurse practitioner	Physicians, nurse practitioners	Working Alliance	Three subscales: bonds, goals, tasks	36 items, 7‐point Likert (adolescent and parent)12 items, 5‐point Likert scale (child)
				

Measurement tool	Related literature	Theoretical foundation of the tool	Internal consistency	Reliability	Measurement error	Content validity	Structural validity	Hypothesis testing	Cross‐cultural validity	Criterion Validity	Responsiveness	Interpretability
Hemofilia‐QoL^32^, ^33^	von Mackensen^68^	Based on the results of qualitative research on the factors that are considered important in quality of life	x	x		x	x	x			x	x
Hemo‐SAT specialist/nurses subscale^31^	von Mackensen,^69^, ^70^ Gringeri^71^	Based on the definition of Weave^72^r: Treatment satisfaction as the individual rating of important attributes of the process and outcomes of his treatment experience	x			x			x			x
Mountain States Patient Satisfaction Survey^34^	NA	Four domains of interest based on literature review of patient satisfaction	x			x						x
Multi‐dimensional health locus of control scale^35^	Wallston^73^	Based on the theory that a person's locus of control has three dimensions, which are independent of one another: internal, external, and luck or chance	x			x						x
QUOTE‐Communication Questionnaire^38^	Sixma,^74^ van den Brink‐Muinen,^75^ Valorie^76^	Based on the conceptual framework for patient satisfaction of Sixma et al^74^ This scale included items from the “Patient Requests Form” of Valorie et al^76^	x			x	x	x	x			x
Theoretical Model of Deliberation Dialogues coding scheme^39^	Bigi^47^	The 3 stages in the “Theoretical Model of Deliberation Dialogues”—opening stage (topic or problem introduced); argumentation stage (solutions proposed, arguments for or against a proposal), closing stage (explicit agreement by one or both participants)		x		x						x
University of Oviedo Survey^36^, ^37^	Chen,^77^ Lee & Lin,^78^ Warren‐Findlow,^79^ Fassaert,^80^ Briggs,^81^ Kriston^82^	Authors considered relational dimensions of communication to design the survey, using items from validated measurement tools: “Diabetes Empowerment Process Scale”,^77^ “Trust in Physician Scale”,^78^, ^83^ “Active Listening Observation Scale”,^77^, ^80^ “Health Literacy Measurement Scale”,^81^ “9‐item Shared Decision Making Questionnaire” 82	x	x		x	x	x				x
Veritas‐PRO communication subscale^40^, ^62^	Duncan,^84^ Lock,^85^ Rubén,^86^ Tran^20^	Development was informed by data gathered from health‐care providers and patient focus groups	x	x		x	x	x	x			x
Wake Forest Trust in Physician Scale^20^	Muller,^87^ Hall,^88^ Bachinger,^89^ Donnelly^90^	Hall et al^88^ conceptualized model focused on interpersonal trust from a patient to a known primary care provider based on a review of theoretical and empirical literature from medical and nonmedical settings	x	x		x	x	x	x			x
Working Alliance Inventory—Chronic Conditions^42^	Besley,^45^ Hall,^43^ Burns,^44^ Morris,^46^ Horvath and Greenberg^60^	This tool was adapted from the “Working Alliance Inventory” in collaboration with its author. The original tool was developed using Bordin's Theory of Working Alliance in psychotherapy, which has three constituent components: bonds (feelings between), goals (valuing the outcomes that are targeted) and tasks (behaviours within the clinical encounter)	x	x	x	x	x	x	x		x	x

a The health‐care discipline for which the tool was developed.

b The interpersonal construct measured by the tool.

### 3.3 Content comparison of the tools

We did not identify any tools that comprehensively measured the full scope of therapeutic relationship. Based on item content analysis, we distinguished six tools that measure a relationship construct as a primary domain: WAI‐CC, WFTPS, QUOTE, Veritas‐PRO, TMDD and UOvS. Three of the four other tools measured satisfaction with care.

The results of our item content analysis showed the WAI‐CC most comprehensively covers the components of therapeutic relationship framework, with 9 of 11 subcomponents represented, missing the subcomponents of “body as a pivot point” and “present.” The scope of the UOvS content was broad as well, capturing 7 of the 11 subcomponents. The WFTPS measured *elements of the bond* (trust, caring) and the *conditions of engagement* (receptive, genuine, committed). The items in the Veritas‐PRO, TMDD and QUOTE tools measured subcomponents of *ways of establishing connections*.

In terms of the three themes of therapeutic relationship, five of the six tools addressed the relationship as a mutual endeavour, and four of six tools addressed the body is central theme. A single tool attended to the personal aspect of therapeutic relationship (UOvS), while all tools examined professional aspects of therapeutic relationship. We compare the six relational tools in terms of functionality, content and measurement properties in Table 4.

**Table 4:** A comparison of the relational tools from the haemophilia literature

Measurement tool	Target population	Purpose of the tool	Content of the tool^a^	Measurement properties^b^	Language
QUOTE‐Communication	Adult and paediatric patients before and after health‐care encounter	To evaluate patient satisfaction with relational communication during an encounter with a health‐care provider, by comparing patient communication expectations and experiences during encounter	“Establishing connections” (acknowledging the individual, body as a pivot point). Themes covered: professional dimension and body is central	Measurement properties have not been reported for a haemophilia population. Evidence of content and construct validity in medical outpatient populations	Dutch, French, Spanish, Flemish, German, English
Theoretical model of deliberation dialogues	Adult patients with haemophilia and health‐care providers	To describe shared decision making during an encounter between a health‐care provider and patient, using an interaction analysis coding scheme	“Establishing connections” (acknowledging the individual). Themes covered: mutuality professional	Content validity, inter‐rater reliability testing, coding scheme was developed in the haemophilia population	Italian
University of Oviedo Survey	Adult patients with haemophilia and health‐care providers	To discriminate between groups of patients with varying levels of different factors related to patient‐provider communication, shared decision making, and empowerment experience	“Establishing connections” (body as a pivot point, giving‐of‐self, acknowledging the individual), “elements of the bond” (trust), and “conditions of engagement” (receptive, committed). Themes covered: mutuality, professional, personal, body is central	Developed and tested in a haemophilia population. Shows evidence of reliability, content and construct validity	Spanish, English (translation not tested)
Veritas‐PRO—”Communication” subscale	Adult and paediatric patients with haemophilia on prophylaxis factor replacement therapy	To evaluate patient‐reported communication behaviours with respect to haemophilia treatment, and quantify change in adherence over time	“Establishing connections” (body as a pivot point). Themes covered: body is central	Developed and tested in a haemophilia population. Evidence of internal consistency, reliability and content validity	English, Dutch, German, Spanish
Wake Forest Trust in physician	Adult patients in medical outpatient and primary care settings and known health‐care provider	To discriminate between patients with varying levels of interpersonal trust towards a known health‐care provider	“Elements of the bond” (trust, caring), and “conditions of engagement” (receptive, genuine, committed). Themes covered: mutuality, professional, body is central	Measurement properties have not been reported for a haemophilia population. Internal consistency, reliability, content and construct validity have been tested in outpatient settings	English, Dutch, German, Spanish
Working Alliance Inventory—Chronic Conditions^c^	Paediatric patients with chronic haematological conditions	To discriminate between patients with varying quality of working alliance with a known physician or nurse practitioner	“Establishing connections” (giving‐of‐self, acknowledging the individual), “Elements of the bond” (trust, caring, respect, rapport), and “conditions of engagement” (receptive, committed, authentic). Themes covered: mutuality, professional	The reliability and usability of this tool were tested in a general paediatric haematology clinic. The measurement properties of the original tool have been tested in the care of adults with diabetes and chronic low back pain	English

a Component(s) of the framework of therapeutic relationship measured by the items in the tool and the themes of therapeutic relationship covered by the tool.

b Measurement properties tested and reported in the literature associated with the tool.

c Content analysis was carried out on the original Working Alliance Inventory—long form because the adapted version was not available.

### 3.4 Outcomes of PPIE

PPIE impacted the study in two specific ways: (a) deciding to use the framework of therapeutic relationship; and (b) informing decisions about the scope of the study. In the design stage, the patient partner considered his experiences during clinical encounters to help us establish the applicability of a framework developed from research in a different patient population to patient‐provider relationships in haemophilia. Also, in early stages, the patient partner was involved in determining the scope of the study. The patient partner actively contributed to writing the project proposal as well as the final manuscript. He supported knowledge dissemination activities by attending scientific conferences where the project was presented and through discussions with peers in his network regarding the project. The patient partner also connected the researchers with other relevant health‐care providers in the community, creating opportunities for future collaboration.

The conception and design of the study and the scope of our research question were guided by informal discussions with health‐care providers working in HTCs. In addition, a peer‐review panel consisting mainly of clinicians from HTCs reviewed the project at the proposal stage, and we incorporated their feedback into the project design.

## 4 DISCUSSION

The purpose of this study was to provide an overview of the measurement of therapeutic relationship in the care of patients with haemophilia. We did not find any studies that measured the full scope of therapeutic relationship. From this, we concluded that no tool for the measurement of therapeutic relationship has been validated in this population.

Knowledge of the performance of a tool in the population of interest is necessary to inform the selection of outcome measures for research applications. The six tools identified in this review show promise as tools to measure subcomponents of therapeutic relationship in haemophilia. However, there is little evidence of the tools' measurement properties from haemophilia patient populations; therefore, further validation of these tools will be required to ensure the results from studies using these tools are valid.

We identified six tools that measure constructs that are part of therapeutic relationship. The features of each tool must be considered when selecting a tool for use in research. The WFTPS may be useful to researchers seeking to measure patient trust in their health‐care provider. It has performed well in studies in outpatient medical settings in both English and Dutch. Similarly, the QUOTE‐communication questionnaire could be used to measure patient satisfaction in studies of relationship‐focused models of care.

The WAI‐CC may be a useful tool to quantify working alliance between health‐care providers and patients with haemophilia. It has been used in the original form in studies of patients with chronic conditions such as low back pain and diabetes.^43^, ^44^, ^45^, ^46^ However, we identified two areas where the content of the tool is incomplete with respect to therapeutic relationship. The first relates to how patients and health‐care providers connect over the body—for example, how physical symptoms are assessed or addressed. This gap in the content of the tool may have significant implications in the care of patients with haemophilia, as a primary concern of patients and health‐care providers is to manage the musculoskeletal manifestations of the condition. The second gap in the content of the WAI‐CC relates to the *“*personal” theme in therapeutic relationship. The study of Vegni et al^15^ revealed a deep personal and professional involvement of haemophilia physicians with their patients,^15^ suggesting that the WAI, which does not address the personal dimensions of therapeutic relationship, may not adequately capture therapeutic relationship in haemophilia. Researchers^43^, ^45^ studying the content of the WAI in physical medicine and rehabilitation have also identified these two limitations.

The items in the UOvS have the potential to be useful in a comprehensive measure of therapeutic relationship. The content of the UOvS subscales is broad, and their measurement properties have been tested in the haemophilia population in Spain. Additionally, there are English and Mandarin translations of most items, which have been tested in populations with chronic conditions. Further measurement studies are needed to adapt the tool to assess therapeutic relationship quality or evaluate change over time.

The reliability and validity of the Veritas‐PRO have been tested in populations of patients with haemophilia. The usefulness of the communication subscale as a measure of therapeutic relationship is uncertain, in part because of the narrow focus of the four items in the scale.

There was one observer‐rating scale identified in this review, a coding schema based on the Theoretic Model of Deliberation Dialogues.^47^ Lamiani et al^39^ reported on the early development and testing stages of an interaction analysis coding for shared decision‐making communication between patients and physicians. The authors anticipate using the tool in a study of factors influencing adherence to treatment in haemophilia.^39^ The coding scheme may be useful in future studies requiring an objective measure of shared decision making during clinical encounters.

### 4.1 Gaps in knowledge and directions for future research

With this scoping review, we identified a need for a valid measure of therapeutic relationship in haemophilia. The first step will be to establish an understanding of the main elements of therapeutic relationship (ie a conceptual model) in the care of patients with haemophilia. This would provide a clear definition and scope of the relational construct being measured by a tool, and would provide a basis for deciding to use an existing tool, from another patient population (ie if the content of an existing tool adequately represents the conceptualization of therapeutic relationship). If an existing tool is not available, the conceptual model would provide a foundation for the development of a new tool.

There are measurement tools developed in other patient populations that could be useful in research with patients with haemophilia. A well‐known tool is the Caring and Relational Empathy (CARE) measure, a 10‐item measure developed for the evaluation of the “human aspects” of the quality of consultations (ie the ability of the health‐care provider to communicate an understanding of the patient's world and to act on that understanding in a therapeutic way).^48^ It has shown good measurement properties in various outpatient settings.^49^ The Healing Encounters and Attitudes Lists (HEAL) is a 57‐item measure of the “patient‐provider connection”.^50^ The HEAL measure has the advantage of being developed using item response theory, which offers greater flexibility and efficiency of measurement.^50^ Eveleigh et al provide an overview of 19 measurement tools that have been used to measure doctor‐patient relationships, but none of these have been tested in patients with haemophilia.^24^

Other considerations for future research include increasing efforts to test and report measurement properties in patients with haemophilia, and studying therapeutic relationship in developing countries. Researchers could make a more informed selection of measurement tools if measurement properties of existing relational tools used in haemophilia populations were known. Also, given the majority of studies we identified were completed in western Europe and North America, studying therapeutic relationship in developing countries should be considered.

This work is important because a validated measurement tool will improve research quality into the processes and mechanisms by which aspects of therapeutic relationship impact outcomes, such as pain, joint health and quality of life for patients with haemophilia. Given that therapeutic relationship is associated with adherence to treatment in haemophilia and that adherence impacts outcomes such as pain and joint health,^19^, ^51^ this a potential area of inquiry that could meaningfully improve the outcomes of care for patients with haemophilia.

### 4.2 Strengths and limitations of this study

We presented a robust overview of research and measurement tools and situate measurement of therapeutic relationship within the broader context of health service research in haemophilia. Also, we identified the knowledge gaps and directions for future research. Some key strengths of our study are that we used a systematic and reproducible search and selection strategy, and we assembled a research team with content and methodological expertise. Further, we clearly reported our approach to data analysis using a robust theoretical framework of therapeutic relationship.

There were two main advantages to using the framework. First, it added structure and transparency to the analysis of the tools' content. The framework was justified given the rigorous methods with which the framework was developed and that therapeutic relationship has not been conceptualized in the care of patients with haemophilia. Second, the framework helped identify a clear distinction between patient‐reported relationship scales and patient satisfaction scales. In an effort to include all available evidence of evaluation of therapeutic relationship, we included patient satisfaction with care as a measurement construct in this scoping study. It was important that we used a method that could distinguish the two constructs, because the use of patient satisfaction questionnaires to evaluate the quality of therapeutic relationships is generally not supported.^52^ In part, this is because general satisfaction questionnaires often fail to include items that assess emotional constructs in the proportions that reflect patients' true priorities in their care.^52^

A potential limitation of the study is that the framework of therapeutic relationship was developed in the context of physiotherapy for patients with musculoskeletal impairments, and the generalizability of the framework from physiotherapy to other health‐care disciplines has not been established. Physiotherapists typically focus on the body and physical condition, and parts of the framework might be more pertinent to physiotherapists (eg “body as pivot point”). However, haemophilia is a haematologic condition that often manifests in the musculoskeletal system. During clinical encounters, health‐care providers from all disciplines will be concerned with asking about physical symptoms, addressing issues related to the physical condition (eg experience of pain, joint bleeding), and how the patient experiences and is impacted by these physical problems. Therefore, the therapeutic relationship framework used is likely relevant to the care of patients with haemophilia by health‐care providers from all disciplines. Furthermore, the framework converges with the therapeutic relationship literature in haemophilia. Qualitative studies in haemophilia addressing a patient‐centred care model^12^ and haematologists' internal representations of difficult encounters with patients^15^ mirror Miciak's^27^ relationship components, as well as the framework's personal and professional theme.

Another potential limitation is the method of appraisal of the content of the items. The process involved the subjective judgement of the researchers, and it is possible that items in each measure would be classified differently by a different set of researchers. In addition, the choice of therapeutic relationship framework could impact the results of the content analysis of the measurement tools. Therapeutic relationship is a complex construct that can be conceptualized and organized differently, thereby impacting the classification of tools as relational. For instance, some frameworks are focused on concepts such as bonding,^53^ empathy,^54^ trust,^55^ or communication,^56^ and working alliance,^30^ while others are more broad, including contextual factors such as the health‐care environment,^57^ patient or health‐care provider factors such as the prerequisite knowledge and qualities of the health‐care provider, or patient expectations for care.^58^, ^59^ Despite these limitations, the results of the content analysis suggest that our method was suitable as there was a clear delineation between the tools classified as relational (proportion of relational items was 0.84 and above) and nonrelational tools (0.38 or lower). A final limitation is that one single researcher conducted the data extraction and content analysis steps; however, these were verified by another researcher.

### 4.3 Reflections on PPIE

The degree of PPIE in health research can range from a consultation‐type involvement to research that is completely led by the public. We engaged a single patient partner who is a graduate student at our institution, who was involved in the early stages (conception, design) and late stages (dissemination). The study could have been enriched by partnering with patients that represent a diversity of backgrounds and experiences or by involving patient partners at all stages of the research process. Despite this limitation, PPIE was an important component of this project, informing principal aspects and leading to a positive learning experience for all involved. The researchers had supportive and open attitudes towards partnering with a patient; however, they were not experienced in the implementation of PPI in practice. We attribute part of the success of PPI in this project to the patient partner's familiarity with research processes, which likely facilitated collaboration. The researchers recognize that a formal mentorship relationship between our research team and a patient‐oriented research organization would be useful in designing and conducting future projects. The aim of the mentorship would be to add structure to the involvement of patient partners, allowing patients who are not already part of the research community to be fully involved in research and to ensure the experience is meaningful for all involved.

## 5 CONCLUSIONS

In this scoping review, we sought to answer the question: “What validated measurement tool(s) exist for measuring the therapeutic relationship in the care of patients with haemophilia?” We did not find any measurement tools that have sufficient validity evidence to be used to measure therapeutic relationship in haemophilia care. We identified six tools that were used to measure aspects of therapeutic relationship, but were not comprehensive in scope. There is a need for a conceptually sound measurement tool of the therapeutic relationship to be validated in the care of patients with haemophilia.

## CONFLICT OF INTEREST

The authors have no conflict of interests to declare.

## AUTHOR CONTRIBUTIONS

E. McCabe, M. Miciak, D. Gross and L. Dennett conducted the literature search; E. McCabe, M. Miciak, D. Gross, L. Dennett, J. Hall, C. Guptill and T. Manns designed the research study; E. McCabe, M. Miciak and D. Gross analysed the data; E. McCabe, M. Miciak and D. Gross contributed to the drafting of the manuscript. All authors critically reviewed and approved the final version of the manuscript.

## DISCLOSURE STATEMENT

EM received financial support for this project through a fellowship grant from the Canadian Hemophilia Society and Shire as well as an operating grant from Bayer Pharmaceuticals, Inc. EM holds a Bayer Hemophilia Awards Program research grant from Bayer Pharmaceuticals, Inc., and has served on advisory boards for Novo Nordisk and Pfizer. MM and DG have research funding from Bayer Hemophilia Awards Program. JH has participated in a leadership programme sponsored by Bayer Pharmaceuticals, Inc. TM, CG and LD have stated that they had no interests that might be perceived as posing a conflict or bias.

## APPENDIX 1

### MEDLINE search strategy

**Table:**

Topic	Population	Measurement instruments
1. professional‐patient relations/ or nurse‐patient relations/	4. exp blood coagulation disorders, inherited/	6. (survey* or tool* or index or test* or instrument* or questionnaire* or scale* or psychometric* or validation or validity or factor analy* or health measurement or health measure or outcome measure or outcome assess* or evaluation).mp
2. ((professional* or doctor* or physician* or nurs* or physiotherap* or physical therap* or social work* or caregiver* or care‐provider* or haemophilia treater* or provider* or hemophilia treater*) adj12 (patient* or client* or consumer* or haemophiliac* or hemophiliac*) adj8 (relation* or relationship* or alliance* or bond or communicat* or encounter* or interaction* or collaboration or trust or empathy or compassion* or responsiveness or caring)).mp. [mp=title, abstract, original title, name of substance word, subject heading word, keyword heading word, protocol supplementary concept word, rare disease supplementary concept word, unique identifier, synonyms]	5. (hemophilia* or haemophilia*).mp	7. “weights and measures”/ or psychometrics/ or questionnaires/
3. (therapeutic alliance* or working alliance* or helping alliance or physiotherapeutic relationship* or therapeutic encounter* or therapeutic process* or patient‐centred* or patient‐centered* or shared decision making or patient satisfaction or quality of care or context* factor*).mp		8. (1 or 2 or 3) and (4 or 5) and (6 or 7)

## APPENDIX 2

### Examples of item content appraisal

**Table:**

Item	Component	Subcomponent	Reasoning
“You have no worries about putting your life in your doctor's hands” [WFTPS]	Elements of the Bond	Trust	The patient trusts the doctor's professional capabilities
“Your doctor will do whatever it takes to get you all the care you need” [WFTPS]	Conditions of Engagement	Committed	The patient believes the health‐care provider to be committed to taking action to help the patient
“I feel our doctor understands us and our problems” [Hemo‐SAT]	Conditions of Engagement	Receptive	The conditions created by the doctor are such that the patient feels understood
“Healthcare professionals help me improve my skills to deal with my illness” [UOv]	Ways of Establishing Connections	Using the body as a pivot point	Describes the information exchange between providers and patients having to do with the illness (physical body)
“The doctor gave me some help with my emotional problems” [QUOTE‐Communication]	Ways of Establishing Connections	Acknowledging the individual	The patient feels that their problems were acknowledged
“I always call the treatment center when I have questions about hemophilia or treatment.” [Veritas‐PRO]	Ways of Establishing Connections	Using the body as a pivot point	Describes accessing/communicating with providers about the disease
“We feel comfortable at the treatment center/hospital” [Hemo‐SAT]	Did not map	‐	Not an interpersonal concept
“I am satisfied with the physiotherapy services” [MSPSS]	Satisfaction with care	‐	Satisfaction with a service
“If I am lucky, my condition will improve” [MHLC]	Did not map	‐	Not an interpersonal concept
“I am genuinely concerned about how (the patient) feels.” [WAI‐CC]	Elements of the Bond	Caring	Describes an emotional investment in the patient's health on the part of the health‐care provider

## APPENDIX 3

### Content analysis of the tools. Results of item appraisal in reference to the components of the therapeutic relationship framework, or “satisfaction with care”

**Table:**

Mapped to the framework (number of items)	Wake Forest Trust in Physician Scale	University of Oviedo survey	Mountain States Patient Satisfaction Survey	QUOTE Questionnaire	Multi‐dimensional health locus of control scale	Veritas‐PRO (communication subscale)	Theoretical model of deliberation dialogues	Hemofilia‐QoL^a^	Hemo‐SAT (physician/nurse subscale)	Working Alliance Inventory—Chronic Conditions^b^
Ways of Establishing Connections	0	10	4	10	4	4	1		0	17
Acknowledging the individual		6	4	4		1	1			15
Giving‐of‐self		1								2
Using the body as a pivot point		3		6	4	3				
Elements of the Bond	6	5	4	0	0	0	0		1	11
Respect			4							4
Trust	5	5							1	3
Caring	1									2
Nature of the rapport										2
Conditions of Engagement	4	9	6	0	0	0	0		1	10
Present			4							0
Receptive	1	3	2						1	7
Genuine	2									1
Committed	1	6								2
Number of items that fit^c^	10	24	14	10	4	4	1	NA	2	32
Number of items in tool	10	29	37	10	18	4	1	NA	7	36
Comprehensiveness										
Proportion of relationship items^e^	1.00	0.83	0.38	1.00	0.22	1.00	1.00	NA	0.29	0.89
Components covered (of 3)	2	3	3	1	2	1	1	NA	1	3
Subcomponents covered (of 11)	5	7	2	2	2	2	1	NA	3	9
Component most often covered	Elements of the bond	Establish connections	NA	Establish connections	NA	Establish connections	Establish connections	NA	NA	Establish connections
Presence of themes^d^										
Mutuality Y/N	Yes	Yes	No	No	Yes	No	Yes	NA	Yes	Yes
Professional Y/N	Yes	Yes	Yes	Yes	Yes	No	Yes	NA	Yes	Yes
Personal Y/N	No	No	No	No	No	No	No	NA	No	No
Body is central Y/N	Yes	Yes	No	Yes	Yes	Yes	No	NA	Yes	No

Notes

a Test items not available, item analysis not possible.

b Item analysis carried out using the original long form of the Working Alliance Inventory, because the adapted version was not available.

c Number of items coded to a component in the framework.

d Determined by whether the content of the tool covers the themes in therapeutic relationship framework.

e Proportion of relationship items over the total number of items in the tool.
